# Linalool vs linalool-loaded chitosan nanoparticles in an Aβ-induced rat model of Alzheimer’s disease: A molecular, biochemical, histological, and behavioral study

**DOI:** 10.22038/ijbms.2025.88638.19143

**Published:** 2025

**Authors:** Mohammad Pakdel, Masoumeh Asle-Rousta, Mehdi Sadegh, Akram Eidi

**Affiliations:** 1 Department of Biology, SR.C., Islamic Azad University, Tehran, Iran; 2 Department of Physiology, Za.C., Islamic Azad University, Zanjan, Iran; 3 Department of Physiology, Faculty of Medicine, Arak University of Medical Sciences, Arak, Iran

**Keywords:** Alzheimer’s disease, Amyloid precursor protein – secretases, Hippocampus, Kynureninem, Linalool-loaded chitosan – nanoparticle, Oxidative stress, Spatial memory

## Abstract

**Objective(s):**

Recent studies have increasingly focused on applying nanotechnology to treat neurodegenerative diseases. In this study, we compared the effects of the monoterpene linalool and linalool-loaded chitosan nanoparticles on key pathological features of Alzheimer’s disease (AD), including oxidative stress, neuroinflammation, neuronal death, amyloid plaque deposition, alterations in tryptophan metabolism, and memory deficit in a rat model of AD.

**Materials and Methods:**

An intracerebroventricular injection of Aβ_42_ (10 µg) was used to induce the AD model. Linalool (25 mg/kg) and nano-linalool (25 mg/kg) were administered orally once daily for 30 consecutive days.

**Results:**

Both linalool and nano-linalool significantly reduced malondialdehyde levels and enhanced superoxide dismutase activity in the hippocampus. They also decreased the mRNA levels of monocyte chemoattractant protein-1, inhibited the up-regulation of beta-secretase, reduced amyloid plaque deposition, and attenuated pyramidal neuron death in the CA1 region. Additionally, treatment with both compounds down-regulated indoleamine 2,3-dioxygenase, lowered kynurenine levels, and increased serotonin concentrations in the hippocampus. Although both treatments improved learning and spatial memory in Aβ-injected rats, nano-linalool’s effectiveness was more significant than that of linalool in modulating the molecular, biochemical, and histological parameters.

**Conclusion:**

Encapsulating linalool in chitosan nanoparticles enhances its effectiveness in improving molecular, biochemical, and histological changes in the hippocampus of rat models of AD.

## Introduction

Alzheimer’s disease (AD) is the leading cause of cognitive impairment in individuals aged 65 and older. It is characterized by the accumulation of amyloid plaques, the formation of neurofibrillary tangles composed of hyperphosphorylated tau protein, and progressive neurodegeneration in various brain regions, including the entorhinal and perirhinal cortex, as well as the hippocampal subfields of the Cornu Ammonis (CA). This neurodegeneration is associated with astrogliosis and increased microglial activity, which releases inflammatory factors. Amyloid beta (Aβ), the primary component of amyloid plaques, plays a crucial role in initiating the glial response. Moreover, a positive correlation exists between this glial response and neurofibrillary degeneration. The loss of neurons and synapses occurs concurrently with tangle formation (1-3). 

Research has shown that the neurotransmission of acetylcholine, glutamate, and serotonin is impaired in Alzheimer’s patients. This impairment contributes to Aβ deposition, an increase in neurofibrillary tangles, and neuronal death, which negatively impacts memory function. For instance, reduced serotonin levels are linked to the production and deposition of Aβ (4, 5). 

Furthermore, a harmful cycle exists involving oxidative stress and Aβ production. Aβ promotes the generation of reactive oxygen species (ROS), which subsequently stimulates the activity of the beta-site amyloid precursor protein cleaving enzyme (BACE), leading to further Aβ production (6). Given Aβ’s critical role in the pathophysiology of AD, extensive research efforts focus on inhibiting Aβ production, reducing its accumulation, and protecting neurons from Aβ toxicity as potential treatments for the disease (7). However, a definitive cure remains elusive.

One of the challenges in drug development for the prevention and treatment of AD is the selective permeability of the blood-brain barrier (BBB). The other significant challenge in treating brain diseases is that many central nervous system drugs must be given in high doses to achieve satisfactory therapeutic efficacy, which leads to severe peripheral side effects. Polymeric nanocarriers present a promising solution to reduce these side effects. They not only help overcome the limitations related to drug transport across the BBB but also enable controlled drug release. Additionally, nanocarriers offer numerous advantages, including high stability, easy production, high encapsulation efficiency, sterilization capability, simple modification with targeting ligands, and a multifunctional nature. Chitosan-based nanoparticles, derived from natural sources, can be readily converted into nanoparticles and possess several benefits, such as biocompatibility, low toxicity, low immunogenicity, surface modification flexibility, and antibacterial properties (8, 9).

Linalool (3,7-dimethyl-1,6-octadien-3-ol) is an acyclic monoterpene that is found abundantly in lavender, sweet basil, bergamot, eucalyptus, and cannabis. After oral administration, linalool is absorbed from the intestine and is primarily metabolized by the liver into free or conjugated forms. It is mainly excreted in urine and, to a lesser extent, in feces (10, 11). Research indicates that brain concentrations of linalool increase significantly after 90 min of inhalation (in comparison to intraperitoneal injection) but decline sharply after 30 min, eventually reaching levels similar to those observed following intraperitoneal administration of this monoterpene (12). Studies have demonstrated that linalool possesses neuroprotective effects (13-15), and there are reports of its protective effects in experimental models of AD (16, 17).

This study compared the effects of linalool and linalool-loaded chitosan nanoparticles on memory impairment, as well as on biochemical, molecular, and histopathological changes in the hippocampus of a rat model of AD. Additionally, the research examined the effects of these compounds on tryptophan metabolism.

## Materials and Methods

### Synthesis of nano-linalool

A solution containing 5 mg of linalool was prepared by dissolving it in dimethyl sulfoxide (DMSO; Sigma-Aldrich, USA) before adding it to a chitosan solution. After thoroughly dissolving and homogenizing the mixture, a sodium tripolyphosphate solution (Sigma-Aldrich, USA) was added dropwise. After one hour, the solution had dissolved entirely, resulting in the formation of nanoparticles. To separate the nanoparticles, centrifugation was performed at 10,000 rpm for one hour. The resulting precipitate was then dissolved in water and stored at -70 °C for future use (18). 

### Animals and grouping

In this experimental study, 60 male Wistar rats weighing between 200 and 220 g were used. The rats were kept under standard laboratory conditions, including a temperature range of 22 to 24 °C, a 12 hr light/dark cycle, adequate ventilation, and unrestricted access to food and water. The animal experiments received approval from the Ethics Committee of the Islamic Azad University - Science and Research Branch (approval number: IR.IAU.SRB.REC.1402.338).

The animals were randomly divided into six groups (each group containing 10 rats). The groups were as follows:

- Control group: Healthy animals that did not undergo surgery or receive any treatment. 

-PBS group: Received phosphate-buffered saline (PBS) as the solvent for Aβ_42_ via intracerebroventricular (ICV) injection, and DMSO as the solvent for linalool, administered for one month. 

- AD group: Aβ_42_ (Sigma-Aldrich, USA) was administered at a dose of 10 μg per rat (5 μg per lateral ventricle) via ICV injection (19). 

- AD.C group: After receiving Aβ_42_ via ICV injection, chitosan was administered orally (200 mg/kg) for one month (20). 

- AD.L group: After receiving Aβ_42_ via ICV injection, linalool (Sigma-Aldrich, USA) was administered orally (25 mg/kg) for one month (21, 22). 

- AD.nL group: After receiving Aβ_42_ via ICV injection, nano-linalool was administered orally (25 mg/kg) for one month.

### Research outline

The animals underwent stereotactic surgery on day 0. Ten days post-surgery, they received treatments with chitosan, linalool, or nano-linalool for 30 consecutive days. The evaluation of the animals’ spatial learning and memory was conducted using the Morris water maze (MWM) test from days 26 to 30 of the treatment period. One day after the treatment concluded, the rats were sacrificed under deep anesthesia with ketamine and xylazine (23), and their hippocampi were extracted. The hippocampus from five rats was used for biochemical analyses, including measurements of serotonin, kynurenine, and malondialdehyde (MDA), as well as superoxide dismutase (SOD) activity. The right hippocampus of the remaining five rats was used for molecular studies, specifically analyzing the expression of BACE1, indoleamine 2,3-dioxygenase 1 (IDO1), and monocyte chemoattractant protein-1 (MCP-1) using real-time PCR. Meanwhile, the left hemisphere of the brain was processed for histological assessment, which involved examining neuronal death in the hippocampus through Nissl staining and assessing amyloid plaque deposition via thioflavin staining.

### Stereotactic surgery

Rats were initially anesthetized using ketamine (50 mg/kg) and xylazine (10 mg/kg). The coordinates for the injection site of Aβ_42_ (5 μg/2 μl) or PBS (2 μl) were determined based on stereotaxic brain coordinates (24). The specific coordinates for the injection site were as follows: anterior-posterior (AP): -0.8 mm, lateral (L): ±1.5 mm, and vertical (V): 3.8 mm. The injection was administered using a Hamilton microsyringe (Hamilton Company, USA) at a rate of 1 μl every 2 min into both lateral ventricles. After completing the infusion, the needle was left in place at the injection site for an additional 2 min.

### Morris water maze

The MWM test consists of two stages. In the first phase, conducted from days 26 to 29 of treatment, the animals were placed in a water tank four times daily. They used cues around the tank to locate a hidden platform submerged 2 cm below the water surface. Data were collected using video tracking software (EthoVision) from Noldus, The Netherlands. The average time (s) taken to reach the platform was recorded each day, serving as a measure of learning. The second phase, known as the probe test, took place on day 30 of treatment to assess spatial memory. During this phase, the platform was removed, and each animal swam in the tank for 60 s. The time spent (s) in the quadrant where the platform had previously been located was recorded, serving as an indicator of memory ability (25). 

### Biochemical investigations

The hippocampi required for biochemical studies were stored at -70 °C. The process began by homogenizing the rat hippocampus in Tris buffer at pH 7.5. The samples were centrifuged at 12,000 g for 20 min. The protein concentration in the supernatant was measured using the Lowry method (26). Finally, we determined the levels of MDA and the activity of SOD.

MDA levels in the hippocampus were measured using the Nalondi™ Lipid Peroxidation Assay Kit (Navand Salamat, Iran), following the kit’s instructions. In brief, MDA reacts with thiobarbituric acid at high temperatures to produce a pink product, which is measured for absorbance at 550 nm. The MDA level is reported as nmol/mg protein.

SOD activity was assessed using the Nasdox™ assay kit (Navand Salamat, Iran). This kit inhibits the oxidation of pyrogallol and measures its oxidation half-life at a specific concentration. SOD activity in an unknown sample is determined at 405 nm by comparing the suppression of pyrogallol oxidation over time with that of a control concentration of SOD. The results are reported as U/mg protein.

Serotonin and kynurenine levels were determined using ELISA kits (MyBioSource, USA) according to the kit instructions. Absorbance was measured at 450 nm, and the results were expressed as nanograms per milligram.

### Real-time PCR

RNA was extracted from the hippocampus using a SinaClon kit (Iran). The extracted RNA was evaluated at 260 nm and the A260/ A280. cDNA was synthesized using the Sinnaclon First Strand cDNA Synthesis Kit (SinaClon, Iran). Real-time PCR was performed to assess gene expression. Each reaction was prepared using 1 μl of primers, 2 μl of cDNA, 6 μl of double-distilled water, and 10 μl of qPCRBIO SyGreen Mix Lo-ROX. To standardize the expression levels of the genes under investigation, we utilized the reference gene glyceraldehyde-3-phosphate dehydrogenase (GAPDH), as its expression remains unchanged in AD brains (27). All tests were conducted three times, and the determination of relative expression for each gene was performed using the 2^–∆∆Ct^ technique (28). Primer 3 software was used to design and verify primers through the NCBI BLAST Tool. The primer sequences are listed in Table 1.

### Histological examinations

The left hemisphere of five rats per group was fixed in 4% paraformaldehyde. After processing the tissue and embedding it in paraffin, five-micrometer sections were prepared from samples taken from the hippocampus, specifically those located between 2.7 mm and 3.7 mm posterior to bregma. Then, the sections were stained with 0.1% cresyl violet **(**Merck**, **Germany) (Nissl staining). For each animal, ten sections of the hippocampus were examined microscopically (25).

For thioflavin-S staining, brain sections were first deparaffinized and hydrated. They were then incubated for 10 to 15 min at room temperature in filtered 1% aqueous thioflavin-S (Sigma-Aldrich, USA). After incubation, the sections were washed three times with distilled water, glycerol, and PBS solution. Finally, a coverslip was placed on the sample for fluorescent photography using an Olympus microscope (Olympus™, Japan) (29).

### Statistical analysis

All data were analyzed using SPSS software version 26.0. The results are presented as mean ± standard error (SE). One-way ANOVA followed by Tukey’s *post hoc* test was employed to detect differences among groups. The significance level was set at *P*<0.05.

### Characterization of nano-linalool

**Figure 1:**
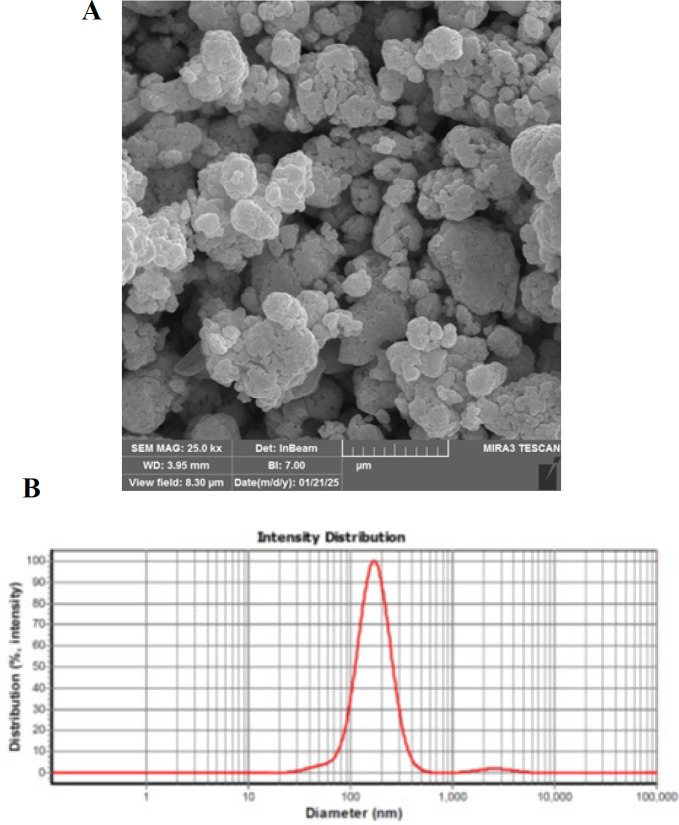
(A) Scanning electron microscopy image and (B) dynamic light scattering analysis for size determination of the nano-linalool

The morphology of nano-linalool was studied using field-emission scanning electron microscopy (FE-SEM), and its particle size was reported to be 159 nm, as measured by dynamic light scattering (DLS) (Figure 1).

### Effect of linalool and nano-linalool on oxidative stress in the hippocampus of Aβ-injected rats

**Figure 2:**
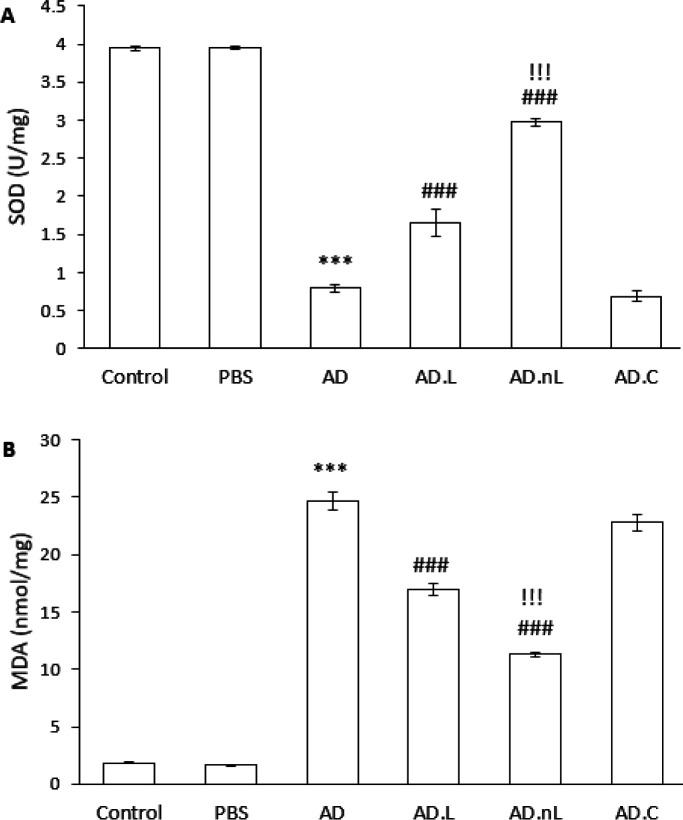
Effect of linalool and nano-linalone on (A) SOD enzyme activity and (B) MDA concentration in the hippocampus of Aβ42-injected rats Results are presented as mean ± SE. (n = 5). *** *P*<0.001 compared to Control group, ### *P*<0.001 compared to AD group and !!! *P*<0.001 compared to the AD.L group (One-way ANOVA). SOD: Superoxide dismutase; MDA: Malondialdehyde

SOD activity was significantly lower in the AD group compared to the Control group (*P*<0.001). However, SOD activity was significantly higher in both the AD.L and AD.nL groups compared to the AD group (*P*<0.001). Additionally, there was a significant increase in SOD activity in the AD.nL group compared to the AD.L group (*P*<0.001). No significant difference in SOD activity was observed between the Control and PBS groups, and there was also no significant difference between the AD.C and AD groups (Figure 2A).

Similarly, the level of MDA was significantly higher in the AD group compared to the Control group (*P*<0.001). In contrast, MDA levels were significantly lower in the AD.L and AD.nL groups compared to the AD group (*P*<0.001). Furthermore, the MDA level in the AD.nL group showed a significant decrease compared to the AD.L group (*P*<0.001). The Control and PBS groups did not exhibit a significant difference, nor was there a considerable difference between the AD.C and AD groups (Figure 2B).

### Effect of linalool and nano-linalool on changes in MCP-1 expression in the hippocampus of Aβ-injected rats

**Figure 3:**
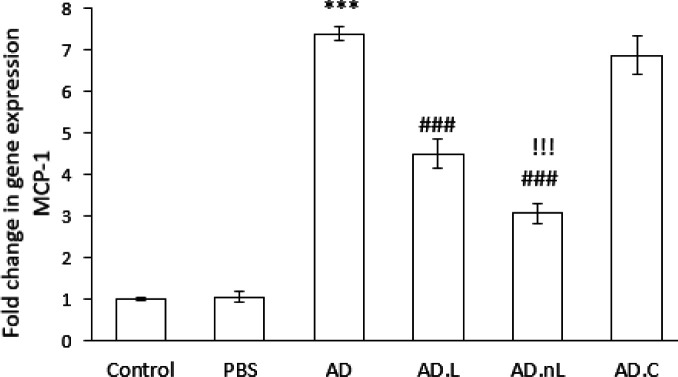
Effect of linalool and nano-linalone on MCP-1 expression in the hippocampus of Aβ42-injected rats

The mRNA expression of MCP-1 in the hippocampus of the AD group was significantly higher than that in the Control group (*P*<0.001). However, these levels were significantly lower in the AD.L and AD.nL groups compared to the AD group (*P*<0.001). Additionally, MCP-1 expression in the AD.nL group showed a significant decrease compared to the AD.L group (*P*<0.001). There was no significant difference in MCP-1 expression between the Control and PBS groups, and no significant difference was observed between the AD.C and AD groups (Figure 3).

### Effect of linalool and nano-linalool on BACE1 expression and Aβ deposition in the hippocampus of Aβ-injected rats

**Figure 4:**
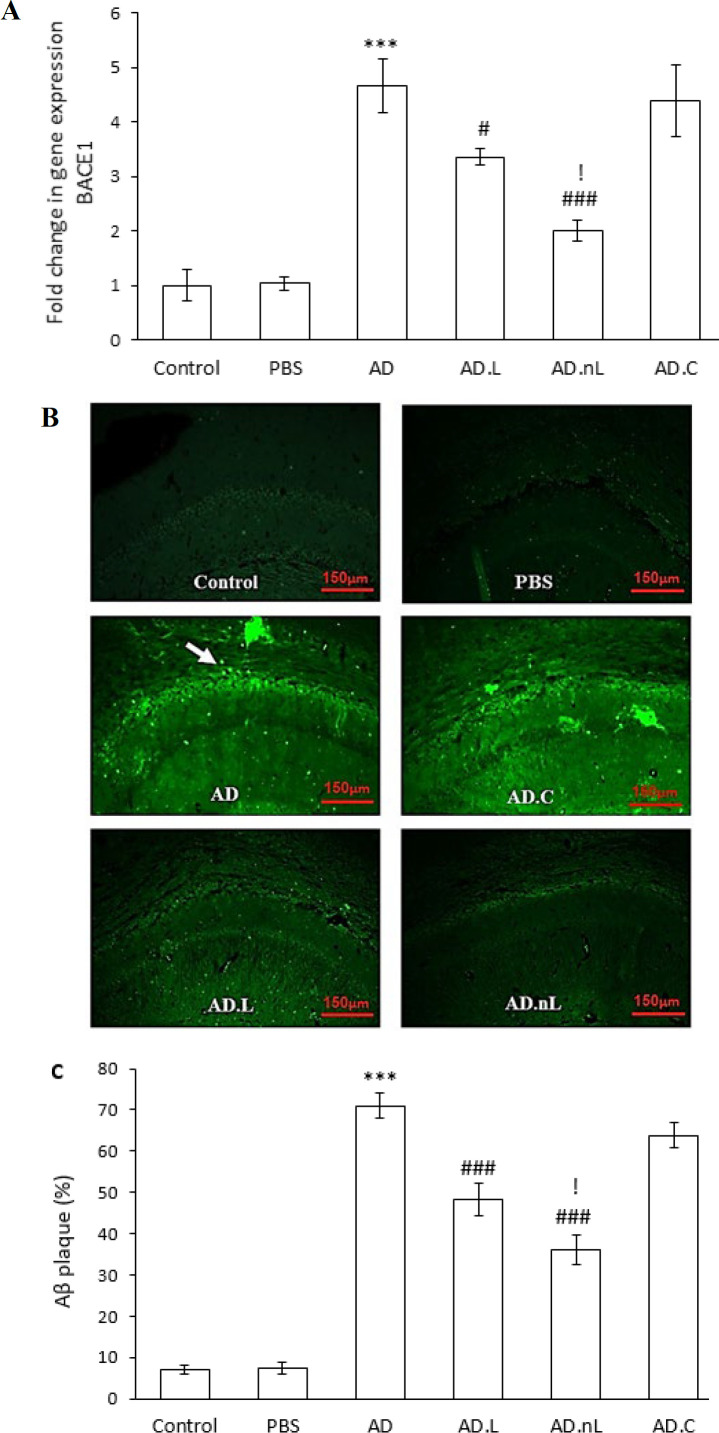
Effects of linalool and nano-linalool on (A) BACE1 expression, (B) amyloid plaque deposition in the CA1 region of the hippocampus of Aβ42-injected rats using thioflavin staining, and (C) statistical comparison of the percentage of amyloid plaque between different groups

The hippocampal expression of BACE1 mRNA was significantly higher in the AD group than in the Control group (*P*<0.001). However, it was meaningfully lower in the AD.L and AD.nL groups compared with the AD group (*P*<0.05 and *P*<0.001, respectively). Additionally, BACE1 expression was significantly lower in the AD.nL group compared to the AD.L group (*P*<0.05). No considerable differences in BACE1 expression were observed between the Control and PBS groups, nor between the AD.C and AD groups (Figure 4A).

Furthermore, a substantial increase in the percentage of Aβ plaque deposition in the CA1 region was observed in the AD group compared to the Control group (*P*<0.001). The percentage of Aβ plaque deposition was remarkably lower in the AD.L and AD.nL groups compared to the AD group (*P*<0.001). The AD.nL group also showed a significant decrease in Aβ plaques compared to the AD.L group (*P*<0.05) (Figures 4B and C). No significant differences were found between the Control and PBS groups or between AD.C and AD groups.

### Effect of linalool and nano-linalool on neuronal death in the CA1 region of the hippocampus of Aβ-injected rats

**Figure 5:**
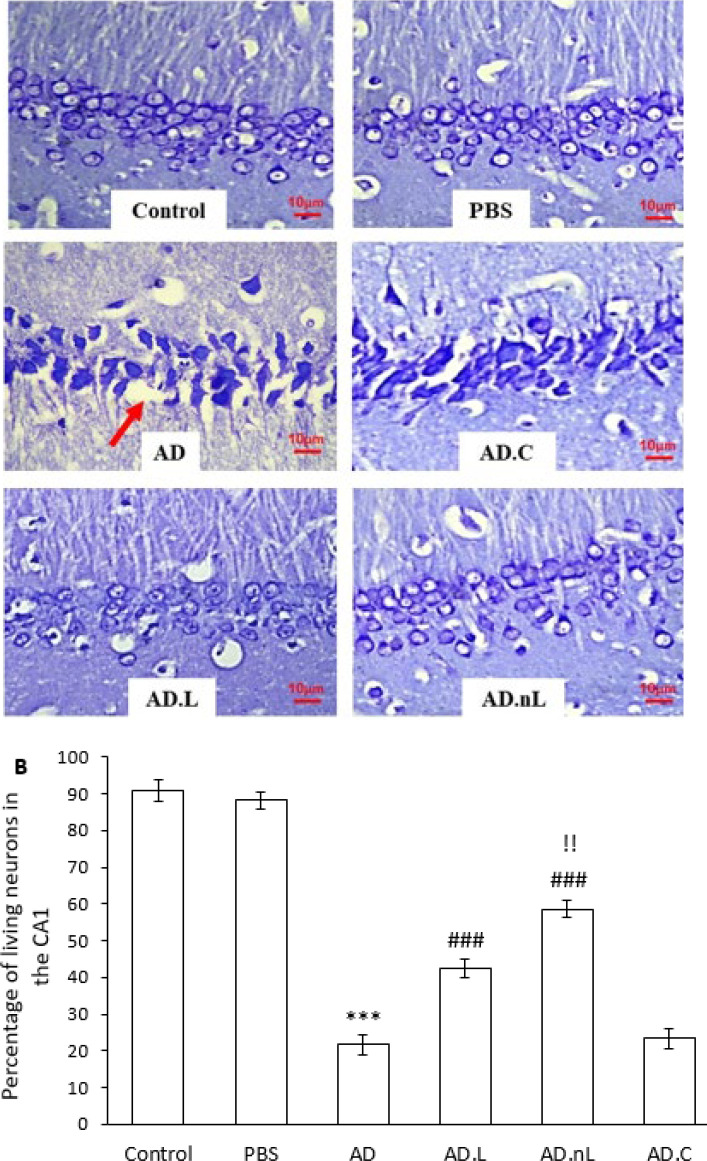
Effect of linalool and nano-linalone on neuronal death in the CA1 region of the hippocampus of Aβ42-injected rats using Nissl staining

Intracerebroventricular injection of Aβ_42_ resulted in significant neuronal death in the CA1 region of the hippocampus. The number of viable neurons in this region was markedly lower in the AD group compared to the Control group (*P*<0.001). Additionally, the pyramidal neuron layer thickness in the CA1 region was reduced and exhibited disruption in some areas. In contrast, the number of viable neurons in both the AD.L and AD.nL groups was significantly higher than in the AD group (*P*<0.001). Furthermore, the AD.nL group showed a significant increase in viable neurons compared to the AD.L group (*P*<0.01) (Figure 5A, B).

### Effect of linalool and nano-linalool on IDO1 expression and kynurenine and serotonin levels in the hippocampus of Aβ-injected rats

**Figure 6:**
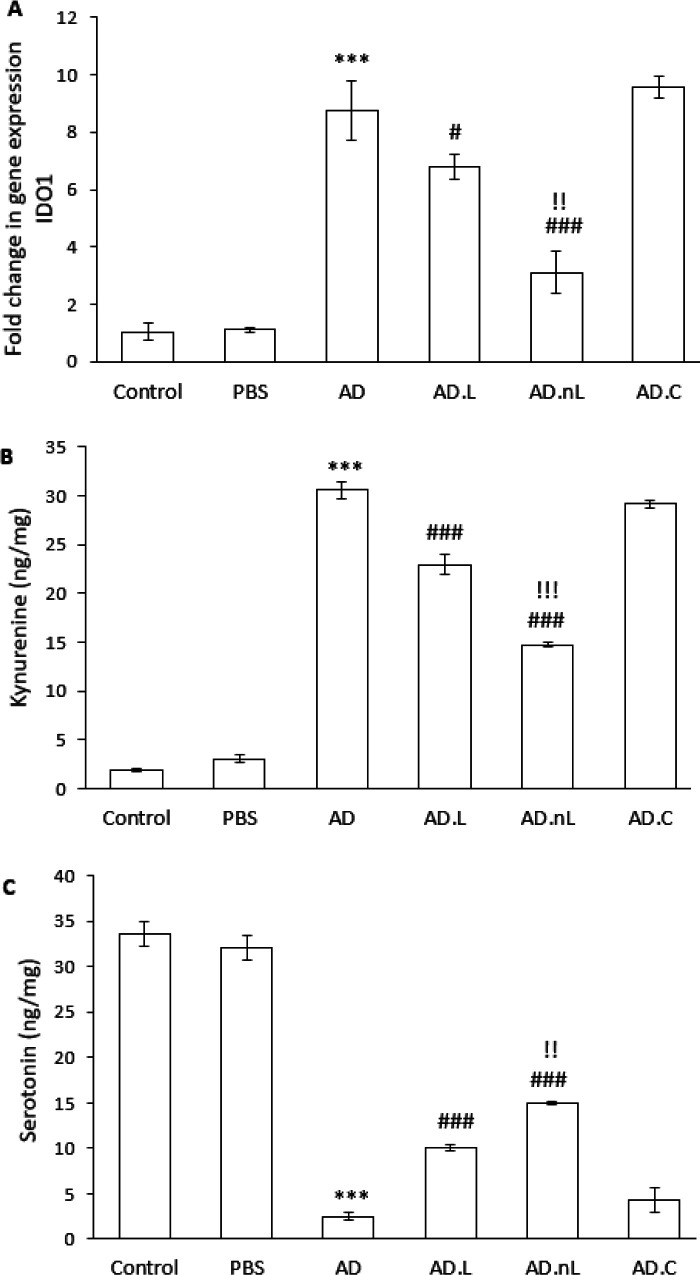
Effects of linalool and nano-linalool on (A) IDO1 expression, (B) kynurenine, and (C) serotonin levels in the hippocampus of Aβ42-injected rats

The mRNA expression of IDO1 in the hippocampus of the AD group was significantly increased compared to the Control group (*P*<0.001). However, this expression was significantly lower in the AD.L and AD.nL groups compared to the AD group (*P*<0.05 and *P*<0.001, respectively). Additionally, the IDO1 expression in the AD.nL group was significantly lower than in the AD.L group (*P*<0.01) (Figure 6A).

Kynurenine levels in the hippocampus of the AD group were significantly higher than those in the Control group (*P*<0.001). Both the AD.L and AD.nL groups showed remarkably lower kynurenine levels compared to the AD group (*P*<0.001), and the AD.nL group had significantly lower levels than the AD.L group (*P*<0.001) (Figure 6B).

The concentration of serotonin in the hippocampus of Aβ-injected rats was significantly reduced. The serotonin level in the AD group was significantly lower than that in the Control group (*P*<0.001). In contrast, the serotonin levels in the AD.L and AD.nL groups were meaningfully higher compared to the AD group (*P*<0.001). Furthermore, the serotonin concentration in the AD.nL group was significantly higher than that in the AD.L group (*P*<0.01) (Figure 6C).

No significant differences were observed between the Control and PBS groups, nor between the AD group and the AD.C group, in any of the factors studied in this section.

### Effect of linalool and nano-linalool on learning and spatial memory deficits in Aβ-injected rats

**Figure 7:**
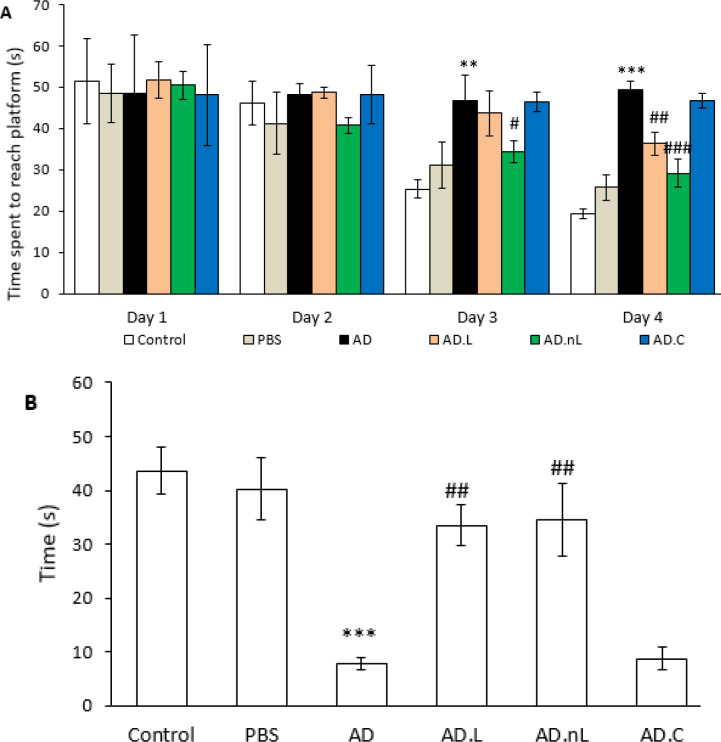
Effects of linalool and nano-linalool on spatial learning and memory in Aβ42-injected rats. Spatial learning and memory were assessed using the Morris water maze

Results from the MWM showed that the AD group performed worse than the Control group in locating the hidden platform. As a result, the AD group spent more time searching for the platform on the third and fourth days of training compared to the Control group (*P*<0.01 and *P*<0.001, respectively). In contrast, the AD.nL group spent less time finding the platform on the third and fourth days compared to the AD group (*P*<0.05 and *P*<0.001, respectively). The AD.L group also spent less time searching on the fourth day than the AD group (*P*<0.01) (Figure 7A). However, there were no significant differences in the performance of the AD.L and AD.nL groups across the different days of testing. In addition, there was no significant difference in the results between the PBS and Control groups or between the AD and AD.C groups. 

In the probe test (platform removed), the AD group spent significantly less time in the target quadrant compared to the Control group (*P*<0.001). However, both the AD.L and AD.nL groups swam for a remarkably longer duration in the target quadrant compared to the AD group (*P*<0.01) (Figure 7B), with no significant difference observed between the AD.L and AD.nL groups. Furthermore, the PBS group showed no significant difference from the Control group, and there was also no notable difference between the AD group and the AD.C group.

## Discussion

In this study, intracerebroventricular injection of Aβ_42_ led to oxidative stress and neuroinflammation in the hippocampus, the deposition of amyloid plaques, and extensive pyramidal neuron death in the CA1 region. Additionally, it caused impaired tryptophan metabolism and negatively affected learning and spatial memory. These findings are consistent with the results of Souza *et al.* (30) and Khan *et al.* (31), indicating that an AD model was effectively induced in rats for this research.

A 30-day treatment with linalool and nano-linalool resulted in a decrease in MDA levels and an increase in SOD activity in the hippocampus of AD model animals. These results are consistent with previous studies, which indicate that linalool can reduce Aβ-induced oxidative stress in the hippocampus (16, 17). Our findings demonstrate that nano-linalool was more effective than linalool in reducing oxidative stress in the hippocampus of animals injected with Aβ_42_. Research has shown that chitosan nanoparticles (without any drug) can protect neuronal cells from oxidative stress-induced death by physically sealing breaches in cell membranes. However, it has been suggested that similar results may not be achievable outside of *in vitro* cultures (32). Given that the MDA levels and SOD activity in the AD.C group did not differ significantly from the AD group, it can be concluded that the improvements observed in the AD.nL group are due to the enhanced anti-oxidant effects of linalool when loaded into chitosan, rather than any contributions from chitosan alone.

Oxidative stress plays a crucial role in the development of AD. There is a bidirectional relationship between oxidative stress and Aβ deposition. A prolonged period of oxidative stress in the brain contributes to the production and accumulation of Aβ through various mechanisms, including the activation of the enzymes BACE and gamma-secretase. Conversely, Aβ exacerbates oxidative stress by stimulating neuroinflammation, activating nuclear factor κB (NF-κB), and inducing mitochondrial dysfunction (33). Our study has shown that linalool and nano-linalool can reduce oxidative stress in the hippocampus of AD model rats. This reduction in oxidative stress is likely to lead to decreased mRNA expression of BACE1 and a lower deposition of amyloid plaques, as evidenced by thioflavin staining. Future research should investigate BACE1 protein levels and activity in the hippocampus of AD model rats treated with linalool and nano-linalool. Additionally, linalool has been found to bind directly to the Aβ peptide, preventing the formation of Aβ fibrils and their aggregation in the rat brain (34). The results of our study indicate that nano-linalool is more effective than linalool alone in reducing BACE1 expression and amyloid plaque deposition. This increased effectiveness may be due to the rapid decrease of linalool levels in the brain (12). In contrast, the encapsulation of linalool in chitosan nanoparticles allows for sustained release, leading to a longer presence of linalool in the brains of animals (35). Therefore, the nano-linalool-chitosan treatment enhances the therapeutic potential of linalool in this context. The lack of measurement of linalool levels in the serum and brain of animals can be considered a limitation of this study.

Oxidative stress leads to the activation of NF-κB, resulting in increased transcription of various genes, including MCP-1. MCP-1 facilitates the recruitment of monocytes to sites of inflammation, leading to the development of inflammation and ultimately exacerbating oxidative stress (36). Both *in vitro* and *in vivo* studies have demonstrated that Aβ induces the up-regulation of MCP-1 (37). Immunohistochemical studies have shown that the expression of NF-κBp65 and MCP-1 is elevated in the hippocampus, as well as in the temporal and frontal regions of patients with AD (38). Sokolova *et al.* (39) identified MCP-1 as the most reliable predictor of AD among 17 cytokines and chemokines studied in the brains of AD patients. Given the reduction in oxidative stress and Aβ deposition in the hippocampus of the AD.L and AD.nL groups, a decrease in MCP-1 expression was also anticipated. Notably, the expression of this chemokine was lower in the AD.nL group compared to the AD.L group. Supporting these findings, Ma *et al.* (40) showed that linalool reduces the expression of MCP-1 and inflammatory cytokines in the lungs by inhibiting the activation of NF-κB. However, a limitation of this study is that we did not assess the expression and activity of NF-κB.

AAD is characterized by severe neuronal death, which significantly exceeds the neuronal loss typically observed in normal brain aging. This neuronal death initially starts in the entorhinal cortex and later occurs in other regions, including the hippocampus, amygdala, temporal lobe, and frontal lobe. Key triggers for neuronal death in AD have been identified as soluble Aβ oligomers, insoluble Aβ aggregates, neurofibrillary tangles, inflammation, and the microglial response (41, 42). Analysis of Nissl-stained hippocampal slices indicated a notable reduction in neuronal loss in the CA1 region among the AD.L and AD.nL groups compared to the AD group. This finding aligns with the reports from Xu *et al.* (16) and Caputo *et al.* (43). Additionally, nano-linalool proved to be more effective in reducing neuronal death in AD model rats than linalool.

Tryptophan metabolism is essential in the development and progression of AD. Certain metabolites of tryptophan have neuroprotective effects and can inhibit Aβ biosynthesis, while others may worsen neuroinflammation and are toxic to neurons, contributing to AD progression. There are two main metabolic pathways for tryptophan: the serotonin pathway and the kynurenine pathway. The serotonin pathway produces serotonin and melatonin, which offer protective effects against various conditions. These products help reduce neuroinflammation, lower Aβ accumulation in the brain, stimulate neurogenesis, prevent hippocampal volume loss, and improve memory in AD models (44, 45). Conversely, the kynurenine pathway is the primary route for tryptophan catabolism, with IDO1 catalyzing the initial, rate-limiting step. IDO1, which is expressed throughout the brain, especially in the hippocampus and limbic system, plays a key role in AD development. Factors like neuroinflammation and intraventricular injection of Aβ_42_ can increase IDO1 expression and activity, leading to more kynurenine production and reduced serotonin synthesis. As a result, IDO is now considered a therapeutic target for AD, as inhibiting this enzyme has been shown to decrease Aβ deposition and improve memory in animal models (30, 46, 47). In this study, linalool and nano-linalool prevented the decrease in serotonin levels, the rise in IDO1 expression, and the increase in kynurenine levels in the hippocampus of Alzheimer’s model rats. This indicates they help maintain normal tryptophan metabolism. An earlier *in vitro* study showed that lavender oil, which contains high amounts of linalool, alpha-pinene, and limonene, significantly reduced IDO activity and kynurenine levels dose-dependently (48). Additionally, linalool can increase serotonin levels in the plasma and hippocampus of sleep-deprived mice, thereby enhancing their learning and memory (49). Considering the notable differences between the AD.L and AD.nL groups in this study, encapsulating linalool in chitosan could improve its effectiveness in regulating tryptophan metabolism. This may be due to better nanoparticle penetration into the brain or extended exposure resulting from its slow, sustained release.

The results of the Morris water maze test revealed significant learning and spatial memory deficits in rats injected with Aβ_42_. In contrast, treatment with linalool and nano-linalool resulted in notable improvements in learning and memory among these animals, aligning with findings from the study conducted by Xu *et al.* (16) on AD model mice. However, it is important to note that conflicting reports exist in this field. For instance, Coelho *et al.* (50) demonstrated that linalool, due to its antagonistic effects on the N-methyl-D-aspartate (NMDA) glutamate receptor, can impair memory acquisition in normal rats. Despite this, since the NR2B subunit of the NMDA receptor is crucial in the progression of AD through calcium influx, antagonists of NR2B are being considered for therapeutic applications in this disease (3, 51, 52). It is recommended that the expression and activity of NMDA receptors in the hippocampus of both AD.L and AD.nL rats be investigated. In another study involving rats subjected to rapid eye movement sleep deprivation, linalool was shown to enhance learning and spatial memory. Researchers attributed this improvement to linalool’s ability to increase serum and hippocampal serotonin levels (49). These findings are consistent with those of our study. Likewise, the inhibitory effect of curcumin-containing nanoparticles, combined with linalool and geraniol, on cholinesterase enzyme activity has also been established (53). This supports the potential of linalool to enhance memory in AD model rats, as cholinesterase inhibitors are recognized therapeutic targets for AD (3). Our study found no significant differences in spatial learning and memory tests between the AD.L and AD.nL groups. Considering that memory is a complex process involving various neurotransmitters that regulate synaptic plasticity (5), we suggest that further investigations into acetylcholine and glutamate neurotransmission in these groups would be valuable.

Some polymeric nanoparticles are stable in blood, non-immunogenic, non-toxic, biocompatible, and non-thrombogenic, but many are not biodegradable, which makes them unsuitable for drug delivery to the brain. Chitosan nanoparticles stand out among polymeric nanocarriers as stable and biodegradable systems for central nervous system drugs (8, 54). For example, the capacity of rivastigmine, one of the most used drugs for treating AD, to reduce oxidative stress, neuroinflammation, and apoptosis in AD model mice was increased when encapsulated with chitosan nanoparticles (55). Based on the results of the present study, encapsulating linalool with chitosan nanoparticles appears to be a suitable option for delivering linalool to the brain, particularly the hippocampus region. Linalool has been shown in several studies to help protect against AD (16, 17, 56). The current study not only confirms those protective benefits but also demonstrates that encapsulating linalool in chitosan enhances its effectiveness. Moreover, the findings indicate that improving tryptophan metabolism significantly contributes to its anti-Alzheimer’s properties. These protective effects were observed following one month of treatment with linalool and nano-linalool. It would be beneficial to investigate longer treatment periods to explore the potential elimination of Aβ-induced damage. Additionally, a similar study is recommended using female rats.

**Table 1:** Primer sequences for the relevant genes were used in the real-time PCR

Reverse primers sequence (5'–3')	Forward primers sequence (5'–3')	Gene
GACCCATTCCTTATTGGGGTCAG	CTCACCTGCTGCTACTCATTCA	MCP-1
ATTCCCGAGCCCACCCATA	ATTTCTCCTGCCCACTCTC	BACE1
CACGAAGTCACGCATCCTC	GCATCAAGACCCGAAAGCAC	IDO1
TGTAGACCATGTAGTTGAGGTCA	AGGTCGGTGTGAACGGATTTG	GAPDH

Results are presented as mean ± SE. (n = 5). *** P<0.001 compared to Control group, ### *P*<0.001 compared to AD group and !!! *P*<0.001 compared to the AD.L group (One-way ANOVA). MCP-1: Monocyte chemoattractant protein-1

White arrows indicate amyloid plaques. Results are presented as mean ± SE. (n = 5). *** *P*<0.001 compared with the Control group, # *P*<0.05 and ### *P*<0.001 compared

with the AD group and ! *P*<0.05 compared with the AD.L group (One-way ANOVA).

BACE: Beta-site amyloid precursor protein cleaving enzyme; CA: Cornu ammonis

(A) A photomicrograph of the CA1 region and the red arrow shows the loss of neuronal layer integrity and layer thinning. (B) Statistical comparison of the percentage of viable pyramidal neurons in the CA1 region in different groups. Results are presented as mean ± SE. (n = 5). *** *P*<0.001 compared with the Control group, ### *P*<0.001 compared with the AD group and !! *P*<0.01 compared with the AD.L group (One-way ANOVA). CA: Cornu ammonis

Results are presented as mean ± SE. (n = 5). *** *P*<0.001 compared to Control group, # *P*<0.05 and ### *P*<0.001 compared to AD group; and !! *P*<0.01 and !!! *P*<0.001 compared to the AD.L group (One-way ANOVA). IDO: Indoleamine 2,3-dioxygenase

(A) Time (s) to reach the hidden platform during four days of training and (B) time (s) spent in the target quadrant in the probe test. Results are presented as mean ± SE. (n = 10). *** P<0.001 and ** P<0.01 compared to Control group and # *P*<0.05, ## *P*<0.01 and ### *P*<0.001 compared to AD group (One-way ANOVA).

## Conclusion

In summary, we concluded that both linalool and linalool loaded into chitosan nanoparticles enhance spatial learning and memory in rats modeled for AD. This improvement is achieved by reducing oxidative stress, inflammation, and neuronal death, while also promoting tryptophan metabolism in the hippocampus. Furthermore, encapsulating this monoterpene into chitosan nanoparticles has amplified its neuroprotective effects, except for memory enhancement.

## Declaration

Grammarly is used to enhance language quality and readability. After using this tool, the author(s) reviewed and edited the content as necessary and take full responsibility for the publication’s content.
